# Plasticity in Major Ampullate Silk Production in Relation to Spider Phylogeny and Ecology

**DOI:** 10.1371/journal.pone.0022467

**Published:** 2011-07-27

**Authors:** Cecilia Boutry, Milan Řezáč, Todd Alan Blackledge

**Affiliations:** 1 Department of Biology and Integrated Biosciences Program, University of Akron, Akron, Ohio, United States of America; 2 Department of Entomology, Crop Research Institute, Prague 6-Ruzyne, Czech Republic; Université de Technologie de Compiègne, France

## Abstract

Spider major ampullate silk is a high-performance biomaterial that has received much attention. However, most studies ignore plasticity in silk properties. A better understanding of silk plasticity could clarify the relative importance of chemical composition versus processing of silk dope for silk properties. It could also provide insight into how control of silk properties relates to spider ecology and silk uses.

We compared silk plasticity (defined as variation in the properties of silk spun by a spider under different conditions) between three spider clades in relation to their anatomy and silk biochemistry. We found that silk plasticity exists in RTA clade and orbicularian spiders, two clades that differ in their silk biochemistry. Orbiculariae seem less dependent on external spinning conditions. They probably use a valve in their spinning duct to control friction forces and speed during spinning. Our results suggest that plasticity results from different processing of the silk dope in the spinning duct. Orbicularian spiders seem to display better control of silk properties, perhaps in relation to their more complex spinning duct valve.

## Introduction

Organisms often exhibit plasticity in their biomechanical traits. For instance, stiffness of insect cuticle [1], strength of plants leaves [2] or the keratin network of cells [3] can vary within one individual in response to environmental changes. Spider silk is noteworthy for both its exceptional performance and its biomechanical variability.

Spider silk is a biopolymer with strong biomimetic potential [4], [5]. It is also central to many aspects of spider ecology, from communication to prey capture. Because silk is so important to spiders, it has presumably been subjected to strong selective pressures during the ∼400 million years of spider evolution [6], [7]. Although modern spiders spin up to seven different types of silk [8], [9], most research focuses on major ampullate silk, particularly forcibly obtained silk (*i.e.* silk manually reeled by a human experimenter from an immobilized spider under controlled conditions). However, this method neglects the potential importance of variation in the properties of major ampullate silk spun under different conditions, especially at the intra-specific level. A clear understanding of silk variability and its mechanisms within a phylogenetic context is needed to understand how spider ecology has shaped the evolution of silk production. This information would also suggest the range of properties that might be achieved in synthetic analogs of spider silk.

Silk structural properties (e.g. diameters of silk threads) and mechanical performance (e.g. failure load) depend upon spinning conditions [10]. Moreover, material properties (*i.e.* the intrinsic qualities of silk) also vary at the inter- and intra-individual levels [11], [12]. Such differences may result in part from variation in amino acid intake [13], [14], constitute a response to prey nutritive value and stimuli [15] and enhance web performance in prey capture [16], [17]. However, the mechanisms of variation in silk material properties remain hypothetical.

Several mechanisms might explain how spiders control silk properties: changes in the chemical composition of the liquid silk dope, variation in the internal environment under which silk dopes are spun into fibers and variation in the external conditions under which fibers are pulled from the spinnerets (see [16] for more details). In orb-weaving spiders, silk is spun from a dope composed of two proteins called MaSp1 and MaSp2 that differ in amino acid sequence [18], [19]. The two proteins likely form different secondary structures [20]–[24]. This should result in different material properties: β-sheets formed by MaSp1 improve silk strength and stiffness while glycine-proline-glycine motifs present in MaSp2 provide elasticity [20], [25]–[29]. Thus, spiders could potentially control the strength and extensibility of major ampullate silk by varying the ratio of MaSp1 to MaSp2 in their glands.

Internal environment within the spider silk gland can influence silk properties. The liquid silk dope stored in the ampulla of the gland passes through the spinning duct before exiting as a solid fiber through the spigot. The liquid dope turns into a solid fiber through various physical and biochemical processes that include pH changes [30]–[32] as well as water and ions re-absorption [31]. Variation in any of these spinning effects could also modulate the material properties of the resulting silk, independently of protein composition.

External spinning conditions also influence silk properties. For instance, spinning speed influences molecular orientation within fibers [33] and thereby material properties, with silk spun at higher rates being stiffer, stronger and less extensible [34], [35]. Friction forces applied when silk is forcibly collected may also increase silk molecular orientation [31] and thereby, silk stiffness [36] and toughness [31].

Here, we focus on three mechanisms by which spiders can modulate silk properties: one biochemical mechanism, and two “spinning effects”. Alteration of the ratio of the two proteins, MaSp1 and MaSp2, that comprise dragline silk, is a possible biochemical mechanism of silk variation. The first “spinning effect” corresponds to changes in silk spinning speed, whether due to spider moving speed or to the speed at which a human experimenter reels dragline. The second “spinning effect” refers to variation in the amount of friction forces applied by the “brake” in the spinning duct. However, these hypotheses focus on evolutionarily derived orb-weaving spiders (Orbiculariae), neglecting the rich evolutionary history of silk production within spiders.

**Figure 1:**
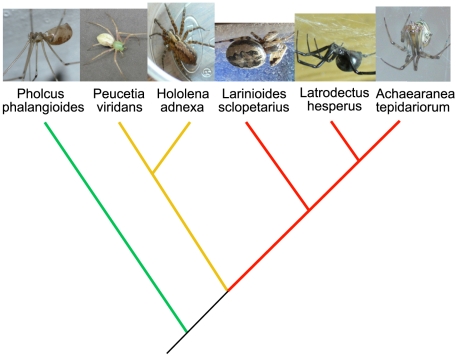
Simple phylogeny of the species used in this study. Haplogynes are in green, RTA clade spiders in yellow and Orbiculariae in red.

In this paper, we consider three major spider clades: haplogynes, RTA clade spiders and Orbiculariae. Haplogynes are among the most basal araneomorph (non-tarantula) spiders and include commonly recognized families such as pholcids (daddy-long-legs spiders), which spin aerial sheet webs. In contrast, entelegynes are far more diverse and abundant. Entelegyne diversity is dominated by two main evolutionary clades, RTA clade spiders and Orbiculariae [37]. Orbiculariae include all orb-weaving species (e.g. garden spiders, barn spiders, golden orb-weavers) as well as many species derived from orb-weaving ancestors, such as the cobweb spinning Theridiidae (widow spiders and their relatives). Finally, the RTA clade includes many different families of spiders that have largely lost the use of silk during prey capture (e.g. lynx spiders, Oxyopidae and jumping spiders, Salticidae), but also some lineages with unique webs such as the terrestrial funnel-web weaving Agelenidae (Fig. 1).

Spiders utilize silk from their major ampullate glands to spin draglines in several ecological contexts. When spiders drop from high positions, they spin a safety line of silk, which prevents them from falling and allows them to climb back to their original position. We refer to it as dropping dragline (DDL). As they walk, spiders also lay a trail of silk, which is used, among other things, for intra-specific communication [38], [39]. We refer to it as walking dragline (WDL). A third type of spinning method, and the most common type of collection for studies of major ampullate silk, is forcible silking. Forcibly obtained silk (which we will call forcible dragline, FDL) is gathered when a human experimenter physically pulls on silk from the spider's spinnerets. These three types of silk differ in spinning speed (e.g. DDL is spun at higher speed than WDL) and friction forces (e.g. some spiders apply high friction forces when spinning FDL but not WDL and DDL).

We define silk plasticity as variation in the material properties of major ampullate silk obtained from a single spider through different spinning methods. To use the terminology defined in the previous paragraph, silk plasticity represents how much the WDL, DDL and FDL spun by a single spider differ in terms of material properties (e.g. strength, stiffness, extensibility, etc.) Despite its potential evolutionary significance, our current knowledge of silk plasticity among spider clades is limited. Previous studies on plasticity focused on Orbiculariae only. Orbiculariae use major ampullate silk in many situations, for instance, in web-building, as a safety line when dropping and as a trail when walking. In contrast, many RTA clade spiders do not build webs, and many of them are terrestrial, so they do not use DDL. Because Orbiculariae make more varied uses of major ampullate silk than RTA clade or haplogyne spiders, higher silk plasticity may be expected in Orbiculariae. However, this has not been tested.

Here, we compare the properties of silk spun under three different conditions (WDL, DDL and FDL) by six species belonging to three diverse evolutionary clades of spiders (Fig. 1): three Orbiculariae species, the cobweb-weavers *Achaearanea tepidariorum* (Theridiidae) and *Latrodectus hesperus* (Theridiidae) and the orb-weaver *Larinioides cornutus* (Araneidae); two RTA clade species, the Agelenidae *Hololena adnexa* and the Oxyopidae *Peucetia viridans*; and finally, one haplogyne species, the Pholcidae *Pholcus phalangioides*.

## Materials and Methods

### 1. Spiders

Five *P. viridans* were purchased from SpiderPharm (Yarnell, AZ, USA). Nine *L. hesperus* were collected from Riverside (CA, USA) and nine *H. adnexa* from Berkeley (CA, USA). Four *P. phalangioides* came from Akron (OH, USA) and four more from Prague (Czech Republic). All other species (five *A. tepidariorum* and twelve *L. cornutus*) were collected from Akron and Bath (OH, USA). All individuals were female, usually adult or subadult. The spiders were housed in the laboratory under a 15∶9 light/dark cycle, at 24°C and fed one to two crickets a week. The spiders were kept in the lab under the same controlled conditions and diet for at least a week before the experiment to minimize any effect of their prior history.

### 2. Silk collection

We collected major ampullate silk using three spinning methods: FDL, DDL and WDL. These three spinning methods correspond to different friction forces applied on the silk and different spinning speeds, maximizing the potential to discover variation in material properties due to spinning effects. We collected four to five samples per individual for each spinning method. The values of the material properties of these four to five samples were tested and then averaged for each individual within spinning method.

To collect FDL, spiders were anesthetized with CO_2_ and restrained on Petri dishes using tape. Silk was manually pulled from the major ampullate spigots at a speed of ∼0.01 m/s and suspended across 15.3 mm gaps on cardboard mounts. The silk was secured to the mount using cyanoacrylate glue. The spider's spinnerets were observed under a dissecting microscope to ensure that the silk collected was produced from the major ampullate spigots. Spiders were awake during silk collection.

To obtain WDL, spiders were placed in a tank lined with a piece of black cardboard folded into fluted 2.5 cm high ridges about 5 cm apart from each other. The spiders then voluntarily laid dragline silk from ridge to ridge as they walked around the enclosure. Unlike forcible silking speed, walking speed was not constant between spiders, and even varied within individual spiders. The silk was collected on cardboard mounts across a 15.3 mm gap, secured with cyanoacrylate glue, and then cut free from the substrate using a hot soldering iron. All samples were composed of two strands of silk of equal diameter, which indicated that they contained only major ampullate threads. Spiders sometimes lay minor ampullate silk along the major ampullate silk as they walk, but these threads are thinner and therefore easily identified. Samples containing minor ampullate silk were discarded.

To obtain DDL, spiders were placed on the border of a ∼1 m high table and gently pushed off the edge after securing a dragline. Dropping spiders typically spun a silk safety line, but were unharmed in the rare instances when they did not. The silk was collected onto a “comb” made of a 75 cm-long strip of balsa wood with ∼10 cm-long “teeth” of balsa glued perpendicularly to it and covered in double-sided tape. This device allowed us to collect many samples of silk, in sequential order, from a single safety line. Once again, silk was collected from the comb onto cardboard mounts across 15.3 mm gaps, secured with cyanoacrylate glue, and then cut free with a soldering iron. Silk may vary along the thread, because the velocity of falling spiders increases and how much they brake changes from the beginning to the end of the fall [40]. Therefore, samples were collected at the beginning, middle and end of the 75 cm-long thread. *P. phalangioides* rarely produced safety lines for the entire 1 m drop. Instead, we pushed these spiders from a height of ∼20–30 cm, and collected only one sample per fall.

The dropping speed of two species (*L. cornutus* and *P. viridans*, n = 2 for each) was estimated using high-speed video. The spiders were placed on top of a 60-cm high box and pushed off the edge until they lowered themselves on a dragline. The falls were recorded at 500 frames/second with a Fastech camera (San Diego, CA, USA). An 8×8 cm cardboard square was used for calibration. ProAnalyst Motion Analysis software (Xcitex, Inc., Cambridge, MA, USA) was used to track the spider's cephalothorax. Dropping speed was calculated as:
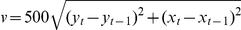
where *y* and *x* are the coordinates of the middle of the spider's cephalothorax, *t* is a given frame and *t-1* is the frame just before *t*.

### 3. Tensile tests and material properties measurements

Silk was placed under a polarized light microscope at 1000x magnification [41] and three pictures of each sample were taken using an Olympus® Q Color5 camera and ImagePro software (Media Cybernetics, Inc., Bethesda, MD). The diameter of each strand was measured three times for each picture using ImageJ (Rasband, W.S., 1997–2009). From the diameter of each strand, we calculated the total cross-sectional area to determine the stress during the tensile tests.

The tensile tests were run on a Nano Bionix (MTS Systems Corp., Oakridge, TN). True stress (σ) represents the force per area exerted on the sample and was calculated assuming constant volume [42] as:
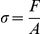
where *F* is the force exerted on the material, and *A* is the instantaneous cross-sectional area of the silk fiber at time *t*.

True strain (*ε*) is the relative extension of the sample and was calculated as:
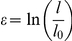
where *l* is the instantaneous length of the fiber at time *t* and *l_0_* is the original length of the fiber. True strain was used instead of engineering strain, since it gives more accurate results for viscoelastic materials [43].

From the stress-strain curves obtained, six material properties were calculated:

Young's modulus measures material stiffness and was calculated as the slope of the stress-strain curve in the elastic, pre-yield region. Stiffness represents how much the material extends if subjected to a given force (pull). Stiffness can have important consequences for silk function. For instance, if spiders spin silk while dropping, the less stiff the silk, the more the thread will extend under a given load.
Yield stress and yield strain were calculated as the stress and strain at yield, respectively. Yield is the transition from elastic to plastic behavior, marking the point after which the material deforms permanently. Thus, yield stress and yield strain represent how much force per area and extension the material can sustain before deforming permanently.
Ultimate strength and extensibility were calculated as the stress and strain at failure, respectively. They represent how much force per area and extension the material can sustain before breaking.
Toughness measures the amount of energy that can be absorbed by the silk before breaking and was calculated as the area under the stress-strain curve. Toughness typically increases with ultimate strength and extensibility. Toughness is critical for silk function because threads are often subjected to high energy impacts during prey capture or when acting as safety lines.

### 4. Statistics

Most material properties are not correlated for major ampullate silk [44]. Therefore, we compared the average silk performance per spider between clades and spinning methods using a full factorial design nested MANOVA with the six material properties as dependent variables and spinning method (FDL, DDL or WDL), spider clade and species as factors. Species was treated as a nested factor within the clade - spinning method interaction. The use of a nested MANOVA with species allowed us to identify possible differences in plasticity between species belonging to the same clade. The MANOVA also identified which properties varied with spinning methods differently for the three clades. For the material properties whose plasticity differed among clades, we assessed how each property differed with spinning method through ANOVAs. We ran a separate ANOVA for each material property and each spider clade (e.g. strength of Orbiculariae silk), with the material property as the dependent variable and spinning method as the independent variable. This series of analyses identified which material properties differed between each pair of spinning methods, and whether these differences were similar between orbicularian, RTA clade and haplogyne spiders. For this series of analyses, once again, we did not average per species and considered each spider and spinning method as an observation. For each material property, the values for the 4–5 samples were averaged.

### Ethics statement

Animals were collected from the University of Akron Field Station under permit 2006–009.

## Results

### 1. Dropping speed of spiders

**Figure 2:**
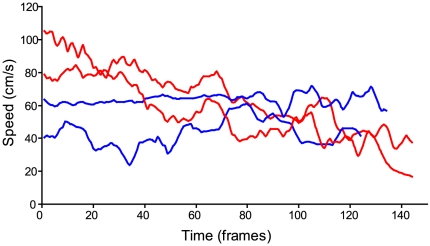
Speed of dropping *Larinioides cornutus* (Orbiculariae, in red) and *Peucetia viridan*s (RTA clade spider, in blue).

*P. viridans* maintained a fairly constant dropping speed of ∼0.05 m/s. In contrast, *L. cornutus* started falling at ∼0.09 m/s before slowing down until less than 0.04 m/s (Fig. 2).

### 2. Do silk properties vary with spinning methods, independently of spider clade?

Table 1 summarizes the average material properties of silk collected by each of the three spinning methods. Silk properties differed between FDL, DDL and WDL when the results from all species were pooled (MANOVA on material properties, effect of spinning method, Wilk's lambda, P<0.0001, n = 134). In particular, compared to WDL, FDL had higher yield stress (Tukey's HSD, P = 0.0028) and yield strain (Tukey's HSD, P = 0.0090) but lower extensibility (Tukey's HSD, P<0.0001) and toughness (Tukey's HSD, P = 0.0022). DDL had intermediate properties: compared to FDL, it had lower yield strain (Tukey's HSD, P = 0.0102), and compared to WDL, it had lower extensibility (Tukey's HSD, P<0.0001).

**Table 1:** Material properties of silk obtained under different spinning conditions from six spider species (average ± SE).

	Young's Modulus (GPa)	Yield Stress (MPa)	Yield Strain (mm/mm)	Ultimate Strength (MPa)	Extensibility (mm/mm)	Toughness (MPa)
*Pholcus phalangioides*						
FDL	10.5±2.8	344±67	0.036± 0.002	1107±215	0.238±0.022	144±29
DDL	11.2±1.0	354±41	0.036±0.003	1054±102	0.199±0.013	117±19
WDL	6.4±0.9	224±25	0.039±0.004	875±60	0.260±0.008	119±8
*Hololena adnexa*						
FDL	10.4±1.7	275±67	0.037±0.007	954±133	0.223±0.032	111±23
DDL	18.9±3.3	367±87	0.026±0.003	1439±124	0.215±0.012	198±34
WDL	20.2±2.1	428±58	0.024±0.003	1906±102	0.245±0.012	260±20
*Peucetia viridans*						
FDL	32.1±1.1	1283±105	0.043±0.003	1738±162	0.126±0.027	157±47
DDL	22.8±2.8	516±18	0.029±0.001	1450±153	0.218±0.021	208±25
WDL	18.9±2.2	497±56	0.030±0.002	1268±86	0.325±0.018	283±43
*Achaearanea tepidariorum*						
DDL	12.1±0.3	314±19	0.029±0.001	1062±144	0.308±0.025	183±42
WDL	9.3±1.0	245±35	0.030±0.001	937±144	0.380±0.027	204±25
*Latrodectus hesperus*						
FDL	18.4±0.9	518±60	0.028±0.001	1552±156	0.340±0.023	293±27
DDL	23.0±1.2	601±33	0.030±0.001	1667±141	0.268±0.019	284±36
WDL	17.3±1.2	449±31	0.030±0.002	1198±144	0.353±0.022	257±18
*Larinioides cornutus*						
FDL	14.8±1.0	516±69	0.040±0.005	1972±65	0.207±0.009	222±13
DDL	17.3±1.6	392±29	0.029±0.002	1768±137	0.293±0.011	278±27
WDL	13.5±0.8	319±19	0.029±0.001	1759±102	0.346±0.010	293±20
All species						
FDL	16.9±1.3	566±59	0.038±0.002	1555±87	0.240±0.014	202±15
DDL	18.0±1.0	433±22	0.030±0.001	1476±67	0.254±0.008	225±15
WDL	14.0±0.9	350±21	0.029±0.001	1370±78	0.310±0.011	238±13

Silk was collected by forcibly silking (FDL), by letting a spider spin while dropping (DDL) and by letting a spider spin while walking (WDL).

### 3. How do silk properties change with spinning methods across different spider clades?

The three clades did not show the same silk plasticity (MANOVA, effect of the interaction of spinning method and valve presence, Wilk's lambda, P = 0.0355, n = 134). Different species from the same clade varied in their plasticity for Young's modulus, yield stress, strength, extensibility (nested MANOVA, effect of species, all P<0.0001, n = 134) and toughness (P = 0.0128). However, despite variability within clades, silk plasticity still differed among the three clades (Orbiculariae, RTA clade and haplogyne) for strength (MANOVA, effect of the interaction of spinning method and valve type, P = 0.0052, n = 134) and toughness (P = 0.0014).

**Figure 3:**
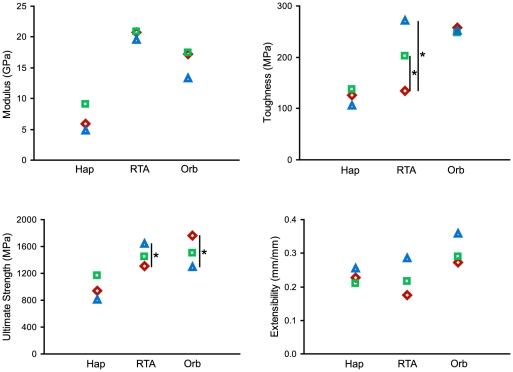
Material properties of FDL (red diamonds), DDL (green squares) and WDL (blue triangles) spun by Haplogynes (Hap), RTA clade spiders (RTA) and Orbiculariae (Orb). Asterisks represent significant differences within a species at α = 0.05.

Both RTA clade spiders and Orbiculariae showed a difference between FDL and WDL, but Orbiculariae had stronger FDL (mean ± SE (MPa), 1762±86 for FDL versus 1499±95 for DDL and 1298±97 for WDL, ANOVA, Wilk's lambda, P = 0.0202, n = 74) while RTA clade spiders had weaker FDL (mean ± SE (MPa), 1346±145 for FDL versus 1444±88 for DDL and 1586±113 for WDL, ANOVA, Wilk's lambda, P = 0.0437, n = 39) (Table 1). Haplogynes showed no difference in strength between silk collected by different methods (ANOVA, Wilk's lambda, P = 0.3794, n = 21). Toughness was not affected by spinning methods for Orbiculariae (ANOVA, Wilk's lambda, P = 0.8746, n = 74) or haplogynes (ANOVA, Wilk's lambda, P = 0.5364, n = 21) but it differed strongly for RTA clade spiders, with both types of naturally spun silk being tougher than FDL (mean ± SE (MPa), 134±21 for FDL versus 203±21 for DDL and 272±19 for WDL, ANOVA, Wilk's lambda, P<0.0001, n = 39) (Figure 3 and Table 1).

## Discussion

### 1. Phylogenetic variation and mechanisms of silk plasticity

The material properties of spider silk we recorded (Table 1) are within the range of values reported in other studies [44]–[46], although *P. viridans* individuals consistently exhibit surprisingly high silk stiffness and yield stress. Silk plasticity was observed in RTA clade and orbicularian spiders, but not in the haplogyne *P. phalangioides*. Plasticity in silk material properties, mainly differences in compliance and strength, was also observed in the orbicularian *Argiope trifasciata* [10]. Differences in silk plasticity between clades can be related to variation in silk biochemical composition and anatomy between haplogyne, RTA clade and orbicularian spiders.

The spinning duct of Orbiculariae includes a well-developed, muscled valve [47], [48], whose role is still debated. The valve is composed of “lips” formed by a thickening of the duct cuticle and is operated by a series of muscles [49]. The valve acts as a clamp that allows spiders to brake when dropping on draglines [48]. This valve may also be the “friction brake” that applies forces during forcible silking [40].

The spinning duct valve became more complex through spider evolution [50]. The valve is present in RTA clade spiders but is most elaborate in Orbiculariae. If complexity is an indication of effectiveness, then orbicularian spiders should have a better functioning valve. Thus, compared to haplogyne and RTA clade spiders, Orbiculariae would be better at resisting forcible silking, thereby applying high friction forces to the silk as it is spun [40]. Such forces increase molecular orientation of the silk fiber, resulting in stronger FDL compared to other spinning methods [36]. This is congruent with our observations.

The valve also allows spiders to control spinning speed during falls. Spinning speed affects silk material properties. During forcible silking, strength and stiffness of silk increase with spinning speed [34], [35]. However, this may be due to the forces generated by the spider as it resists silking more when reeling speed increases, rather than speed *per se*. When dissolved silk proteins are spun *in vitro*, stiffness and ultimate strength also increase with drawing speed both in spiders [51], [52] and in silkworms [53]. This suggests that higher spinning speed *per se* results in stronger and stiffer silk. In this case, Orbiculariae's ability to control spinning speed during falls would allow them to spin DDL and WDL at similar speed, resulting in similar properties, as observed here. The fact that spiders brake as they fall is supported by the fact that *Larinioides cornutus* speed decreasesduring falls. Friction forces are also applied when the spider brakes during a fall, but the forces applied during falls are much smaller than those applied during forcible silking, and probably do not increase silk molecular orientation significantly [40].

We hypothesized that the reduced valve of RTA clade and the lack of a valve in haplogyne spiders would not allow them to resist forcible silking or control speed during falls as much as Orbiculariae. In that case, we should see no differences in the properties of FDL and WDL, as was the case here. However, RTA clade and haplogyne spiders should also spin DDL at higher speed than WDL, if they cannot control their speed during drops. This should result in DDL that is stiffer and stronger than WDL, which was not the case in our experiment. This suggests these clades utilize alternative mechanisms to control their dropping speed, possibly using their hindlegs to slow down (pers. obs.). It is also possible that even a reduced spinning duct valve functions as a brake during falls, but not as a friction brake to resist forcible silking. Accordingly, *Peucetia viridans* maintained a constant dropping speed instead of accelerating.

Nevertheless, Orbiculariae, RTA clade and haplogyne spiders differ in much more than just the complexity of their spinning duct valves. For instance, Orbiculariae express two different silk proteins (MaSp1 and MaSp2) in their major ampullate glands [18], [19]. Although data are sparse, haplogyne and RTA clade spiders probably lack these two well-differentiated proteins and instead produce only MaSp1-like proteins [54]–[56]. The histology of the glands' epithelium and general anatomy of the spinning apparatus also differ between clades [57]. Therefore, variation in the material properties of silks spun under different conditions may also result from changes in silk protein composition or aspects of the morphology of the spinning apparatus other than the valve itself. In particular, if changes in the proportion of the two silk proteins, MaSp1 and MaSp2, determine differences in silk properties, then RTA clade spiders should not exhibit any variability since they only possess proteins that are quite close in structure. Yet, RTA clade spiders do show silk plasticity (FDL differed from WDL in RTA clade species). This supports our hypothesis that silk plasticity is due to “spinning effects” more than biochemical changes.

### 2. Functional consequences of silk plasticity: Relation between silk control and silk uses

The orbicularian spiders we studied could control silk properties by regulating their dropping speed and applying friction forces to the thread. In contrast, the RTA clade spiders did not resist forcible silking and the only haplogyne species studied showed no plasticity at all. These results suggest that Orbiculariae can control silk properties better than RTA clade and haplogyne spiders, although a larger sample of spider species is needed to confirm this hypothesis. Major ampullate silk is used by spiders to perform many behaviors, but we argue that derived spiders, such as Orbiculariae, use major ampullate silk for a greater diversity of functions than other clades, such as RTA clade or haplogyne spiders. For instance, many RTA clades species do not spin webs, unlike Orbiculariae. Even when RTA clade or haplogyne spiders spin webs, these webs are less complex and stereotyped than those of Orbiculariae [37]. Orbicularian webs are made of clearly distinct elements composed of major ampullate silk, unlike webs from other clades that lack aerial frameworks defining overall web structure. All this suggests that Orbiculariae make more complex use of their major ampullate silk than RTA clade or haplogyne spiders.

Improved control of silk properties may have been selected for as spiders diversified uses of their silk in order to allow spiders to tune silk properties to silk intended use. For instance, in *Achaearanea tepidariorum* cobweb, the vertical threads used for prey capture are more compliant than the horizontal threads used for support, even though both are composed of major ampullate silk [12]. It is possible that *Achaearanea* spiders control the spinning speed and friction forces applied to their silk thanks to their spinning duct valve, such that the material properties of the silk composing the different web regions vary.

Forcibly obtained silk properties differ from naturally spun silk in both RTA clade spiders and Orbiculariae. This is consistent with other studies [59]–[61]. Therefore, when forcible dragline is used to characterize the properties of a species silk, it may not be representative of silk spun *in natura* by the spider, except maybe if the silk is reeled very slowly. If silk is collected while the spider is under anesthesia, the animal cannot brake and apply friction forces to the silk. On the other hand, acidification of the hemolymph and silk dope due to the CO_2_ used in anesthesia may also affects silk properties [60]. Therefore, studies interested in relating silk properties to spider ecology (for instance, how silk is used in webs as in [62]–[64] or how silk varies across species, as in [44]), collecting silk spun by walking spiders may be a better option than collecting silk by forcible silking.

We showed that silk plasticity, measured as variability in the material properties of silk spun under different conditions (FDL, DDL and WDL), exists in several groups of entelegyne spiders. We found strong evidence that silk plasticity is associated primarily with forces applied to the silk within the duct, and not with biochemical changes, since biochemical changes do not agree with our observation of plasticity in RTA clade spiders. Thus, spinning conditions are critical determinants of spider silk material properties. In particular, the presence of a complex spinning duct valve in Orbiculariae seems related to the ability of spiders to control silk properties. This improved control of silk properties may have allowed Orbiculariae to use major ampullate silk in more diverse ways.

## References

[1] Vincent JFV (2002). Arthropod cuticle: a natural composite shell system.. Composites Part A-Applied Science And Manufacturing.

[2] Read J, Stokes A (2006). Plant biomechanics in an ecological context.. American Journal of Botany.

[3] Wöll S, Windoffer R, Leube RE (2007). p38 MAPK-dependent shaping of the keratin cytoskeleton in cultured cells.. Journal of Cell Biology.

[4] Eadie L, Ghosh TK (2011). Biomimicry in textiles: Past, present and potential. An overview.. Journal of the Royal Society Interface.

[5] Kluge JA, Rabotyagova U, Leisk GG, Kaplan DL (2008). Spider silks and their applications.. Trends in Biotechnology.

[6] Craig CL (2003). Spiderwebs and silk: Tracing evolution from molecules to genes to phenotypes..

[7] Sensenig A, Agnarsson I, Blackledge TA (2010). Behavioural and biomaterial coevolution in spider orb webs.. Journal of Evolutionary Biology.

[8] Blackledge TA, Hayashi CY (2006). Silken toolkits: Biomechanics of silk fibers spun by the orb web spider *Argiope argentata* (Fabricius 1775).. Journal of Experimental Biology.

[9] Hinman MB, Jones JA, Lewis RV (2000). Synthetic spider silk: A modular fiber.. Trends in Biotechnology.

[10] Garrido MA, Elices M, Viney C, Pérez-Rigueiro J (2002). Active control of spider silk strength: Comparison of dragline spun on vertical and horizontal surfaces.. Polymer.

[11] Madsen B, Shao Z, Vollrath F (1999). Variability in the mechanical properties of spider silks on three levels:interspecific, intraspecific and intraindividual.. International Journal of Biological Macromolecules.

[12] Boutry C, Blackledge TA (2009). Biomechanical variation of silk links spinning plasticity to spider web function.. Zoology.

[13] Zax DB, Armanios DE, Horak S, Malowniak C, Yang Z (2004). Variation of mechanical properties with amino acid content in the silk of *Nephila clavipes*.. Biomacromolecules.

[14] Tso I-M, Shu-Ya Chiang S-Y, Blackledge TA (2007). Does the giant wood spider *Nephila pilipes* respond to prey variation by altering web or silk properties?. Ethology.

[15] Blamires SJ, Chao IC, Tso IM (2010). Prey type, vibrations and handling interactively influence spider silk expression.. Journal of Experimental Biology.

[16] Boutry C, Blackledge TA (2008). The common house spider alters the material and mechanical properties of cobweb silk in response to different prey.. Journal of Experimental Zoology A.

[17] Tso I-M, Wu H-C, Hwang I-R (2005). Giant wood spider *Nephila pilipes* alters silk protein in response to prey variation.. Journal of Experimental Biology.

[18] Xu M, Lewis RV (1990). Structure of a protein superfiber: Spider dragline silk.. Proceedings of the National Academy of Science U S A.

[19] Hinman MB, Lewis RV (1992). Isolation of a clone encoding a second dragline silk fibroin.. Journal of Biological Chemistry.

[20] Hayashi CY, Shipley NH, Lewis RV (1999). Hypotheses that correlate the sequence, structure, and mechanical properties of spider silk proteins.. International Journal of Biological Macromolecules.

[21] Kümmerlen J, van Beek JD, Vollrath F, Meier BH (1996). Local structure in spider dragline silk investigated by two-dimensional spin-diffusion nuclear magnetic resonance.. Macromolecules.

[22] Liivak O, Flores A, Lewis R, Jelinski LW (1997). Conformation of the polyalanine repeats in minor ampullate gland silk of the spider *Nephila clavipes*.. Macromolecules.

[23] Rising A, Nimmervoll H, Grip S, Fernandez-Arias A, Storckenfeldt E (2005). Spider silk proteins - Mechanical property and gene sequence.. Zoological Science.

[24] van Beek JD, Hess S, Vollrath F, Meier BH (2002). The molecular structure of spider dragline silk: Folding and orientation of the protein backbone.. Proceedings of the National Academy of Science U S A.

[25] Gosline JM, Demont ME, Denny MW (1986). The structure and properties of spider silk.. Endeavour.

[26] Gosline JM, Guerette PA, Ortlepp CS, Savage KN (1999). The mechanical design of spider silks: From fibroin sequence to mechanical function.. Journal of Experimental Biology.

[27] Guerette PA, Ginzinger DG, Weber BHF, Gosline JM (1996). Silk properties determined by gland-specific expression of a spider fibroin gene family.. Science.

[28] Savage KN, Gosline JM (2008). The role of proline in the elastic mechanism of hydrated spider silks.. Journal of Experimental Biology.

[29] Simmons AH, Michal CA, Jelinski LW (1996). Molecular orientation and two-component nature of the crystalline fraction of spider dragline silk.. Science.

[30] Dicko C, Vollrath F, Kenney JM (2004). Spider silk protein refolding is controlled by changing pH.. Biomacromolecules.

[31] Vollrath F, Knight DP (2001). Liquid crystalline spinning of spider silk.. Nature.

[32] Gaines WA, Sehorn MG, Marcotte WR (2010). Spidroin N-terminal domain promotes a pH-dependent association of silk proteins during self-assembly.. Journal of Biological Chemistry.

[33] Eles PT, Michal CA (2004). A DECODER NMR study of backbone orientation in *Nephila clavipes* dragline silk under varying strain and draw rate.. Biomacromolecules.

[34] Chen X, Shao ZZ, Vollrath F (2006). The spinning processes for spider silk.. Soft Matter.

[35] Vollrath F, Madsen B, Shao ZZ (2001). The effect of spinning conditions on the mechanics of a spider's dragline silk.. Proceedings of the Royal Society of London Series B-Biological Sciences.

[36] Perez-Rigueiro J, Elices M, Plaza G, Real JI, Guinea GV (2005). The effect of spinning forces on spider silk properties.. Journal of Experimental Biology.

[37] Blackledge TA, Scharff N, Coddington JA, Szuts T, Wenzel JW (2009). Reconstructing web evolution and spider diversification in the molecular era.. Proceedings of the National Academy of Sciences of the United States of America.

[38] Tietjen WJ, Rovner JS, Witt PN, Rovner JS (1983). Chemical communication in lycosids and other spiders.. Spider communication mechanisms and ecological significance.

[39] Pollard SD, MacNab AM, Jackson RR, Nentwig W (1987). Communication with chemicals: pheromones and spiders.. Ecophysiology of spiders.

[40] Ortlepp CS, Gosline JM (2004). Consequences of forced silking.. Biomacromolecules.

[41] Blackledge TA, Cardullo RA, Hayashi CY (2005). Polarized light microscopy, variability in spider silk diameters, and the mechanical characterization of spider silk.. Invertebrate Biology.

[42] Guinea GV, Pérez-Rigueiro J, Plaza GR, Elices M (2006). Volume constancy during stretching of spider silk.. Biomacromolecules.

[43] Blackledge TA, Swindeman JE, Hayashi CY (2005). Quasistatic and continuous dynamic characterization of the mechanical properties of silk from the cobweb of the black widow spider *Latrodectus hesperus*.. Journal of Experimental Biology.

[44] Swanson BO, Blackledge TA, Beltran J, Hayashi CY (2006). Variation in the material properties of spider dragline silk across species.. Applied Physics A-Materials Science & Processing.

[45] Lawrence BA, Vierra CA, Moore AMF (2004). Molecular and mechanical properties of major ampullate silk of the black widow spider, *Latrodectus hesperus*.. Biomacromolecules.

[46] Shao ZZ, Vollrath F (1999). The effect of solvents on the contraction and mechanical properties of spider silk.. Polymer.

[47] Vollrath F (2000). Strength and structure of spiders' silks.. Reviews in Molecular Biotechnology.

[48] Vollrath F, Knight DP (1999). Structure and function of the silk production pathway in the Spider *Nephila edulis*.. International Journal of Biological Macromolecules.

[49] Wilson RS (1962). The structure of the dragline control valves in the garden spider.. Quarterly Journal of Microscopical Science.

[50] Wilson RS (1969). Control of drag-line spinning in certain spiders.. American Zoologist.

[51] Lazaris A, Arcidiacono S, Huang Y, Zhou J-F, Duguay Fo (2002). Spider silk fibers spun from soluble recombinant silk produced in mammalian cells.. Science.

[52] Seidel A, Liivak O, Calve S, Adaska J, Ji G (2000). Regenerated spider silk: Processing, properties, and structure.. Macromolecules.

[53] Capello J, McGrawth KP, Kaplan D, Adams WW, Farmer B, Viney C (1994). Spinning of protein polymer fibers.. Silk Polymers: Materials Science and Biotechnology (ACS Symposium Series).

[54] Pouchkina-Stantcheva NN, McQueen-Mason SJ (2004). Molecular studies of a novel dragline silk from a nursery web spider, *Euprosthenops* sp. (Pisauridae).. Comparative Biochemistry and Physiology B: Biochemistry & Molecular Biology.

[55] Rising A, Johansson J, Larson G, Bongcam-Rudloff E, Engström W (2007). Major ampullate spidroins from *Euprosthenops australis*: Multiplicity at protein, mRNA and gene levels.. Insect Molecular Biology.

[56] Tian M, Liu C, Lewis R (2004). Analysis of major ampullate silk cDNAs from two non-orb-weaving spiders.. Biomacromolecules.

[57] Kovoor J (1977). La soie et les glandes séricigènes des arachnides.. L'Année Biologique.

[58] Liu Y, Sponner A, Porter D, Vollrath F (2008). Proline and processing of spider silks.. Biomacromolecules.

[59] Guinea GV, Elices M, Real JI, Gutierrez S, Perez-Rigueiro J (2005). Reproducibility of the tensile properties of spider (*Argiope trifasciata*) silk obtained by forced silking.. Journal of Experimental Zoology Part A-Comparative Experimental Biology.

[60] Madsen B, Vollrath F (1999). Mechanics and morphology of silk drawn from anesthetized spiders.. Naturwissenschaften.

[61] Perez-Rigueiro J, Elices M, Llorca J, Viney C (2001). Tensile properties of *Argiope trifasciata* drag line silk obtained from the spider's web.. Journal of Applied Polymer Science.

[62] Craig CL (1987). The ecological and evolutionary interdependence between web architecture and web silk spun by orb web weaving spiders.. Biological Journal of the Linnean Society.

[63] Ko FK, Jovicic J (2004). Modeling of mechanical properties and structural design of spider web.. Biomacromolecules.

[64] Opell BD (1994). Increased stickness of prey capture threads accompanying web reduction in the spider family Uloboridae.. Functional Ecology.

